# A novel, jitter-based method for detecting and measuring spike synchrony and quantifying temporal firing precision

**DOI:** 10.1186/2042-1001-2-5

**Published:** 2012-05-02

**Authors:** Ariel Agmon

**Affiliations:** 1 Department of Neurobiology and Anatomy and the Sensory Neuroscience Research Center, West Virginia University, Morgantown, WV, 26506-9303, USA

## Abstract

**Background:**

Precise spike synchrony, at the millisecond or even sub-millisecond time scale, has been reported in different brain areas, but its neurobiological meaning and its underlying mechanisms remain unknown or controversial. Studying these questions is complicated by the lack of a validated, well-normalized and robust index for quantifying synchrony. Previously used measures of synchrony are often improperly normalized and thereby are not comparable between different experimental conditions, are sensitive to variations in firing rate or to the firing rate differential between the two neurons, and/or rely on untenable assumptions of firing rate stationarity and Poisson statistics. I describe here a novel measure, the Jitter-Based Synchrony Index (JBSI), that overcomes these issues.

**Results and discussion:**

The JBSI method is based on the introduction of virtual spike jitter. While previous implementations of the jitter method used it only to detect synchrony, the JBSI method also quantifies synchrony. Previous implementations of the jitter method used computationally intensive Monte Carlo simulations to generate surrogate spike trains, whereas the JBSI is computed analytically. The JBSI method does not assume any specific firing model, and does not require that the spike trains be locked to a repeating external stimulus. The JBSI can assume values from 1 (maximal possible synchrony) to −1 (minimal possible synchrony) and is therefore properly normalized. Using simulated Poisson spike trains with introduced controlled spike coincidences, I demonstrate that the JBSI is a linear measure of the spike coincidence rate, is independent of the mean firing frequency or the firing frequency differential between the two neurons, and is not sensitive to co-modulations in the firing rates of the two neurons. In contrast, several commonly used synchrony indices fail under one or more of these scenarios. I also demonstrate how the JBSI can be used to estimate the spike timing precision in the system.

**Conclusions:**

The JBSI is a conceptually simple and computationally efficient method that can be used to compute the statistical significance of firing synchrony, to quantify synchrony as a well-normalized index, and to estimate the degree of temporal precision in the system.

## Background

The most basic temporal relationship in the active nervous system is firing synchrony, that is, the coincidence of spikes fired by two or more neurons. Firing synchrony has been widely reported in the nervous system and is of much interest to neuroscientists both because of its potential role in encoding, transmitting and decoding of information in the brain, and because it may reveal something about the underlying synaptic (or other) interactions that generate it (for reviews, see [1,2]). Firing synchrony increases the reliability of firing in downstream neurons that receive the coincident synaptic inputs [3-5] and may therefore be an efficient way to transmit information through multiple layers of feed-forward excitatory networks [6-8]. Alternatively or in addition, synchrony may be used as a ‘binding’ signal [9] (but see [10]); may be the neural correlate of attention or expectation [11-13]; or may encode information about the stimulus, beyond that available from the firing rate alone [14,15]. Synchrony can arise when two or more neurons respond to a sudden onset or rapid modulation of a sensory stimulus, such as an auditory click [16], a visual saccade [17] or a high-velocity whisker deflection [18-20]. The focus here, however, will be on stimulus-independent synchrony that is observed (in some systems) after correcting for stimulus effects, or in the absence of a stimulus [5,21,22]. Whereas stimulus-dependent synchrony may encode information about the stimulus that drives it, stimulus-independent synchrony may teach the experimenter something about the connectivity of the underlying network. Two network motifs have most often been invoked to account for stimulus-independent synchrony: shared excitatory inputs from diverging presynaptic axons [23,24] and electrical coupling by gap junctions [25-27]. In a recent publication, we demonstrated that a third motif, reciprocal inhibitory chemical synapses, can drive stimulus-independent synchrony with sub-millisecond precision, in the absence of shared inputs or electrical coupling [28].

Since spike coincidence could also be a purely chance occurrence, an important first step in studying synchrony is to determine if the observed coincidence level is ‘real’, meaning statistically significant - in other words, to calculate its *p*-value or *Z*-score. We refer to this as detecting synchrony. However, if one wants to decipher the synaptic connectivity driving the synchrony, one needs to go beyond a *p*-value and to quantify the synchrony by a well-normalized ‘synchrony index’, which can be examined for correlation with synaptic parameters such as the strength of the electrical coupling or the rate of shared inputs. Various methods for achieving the twin goals of detecting and quantifying synchrony, some more practical than others, have been proposed and used in the past, and some of them are described and examined in detail in a recently published compendium [29]. Unfortunately, many previously proposed methods have serious limitations when used for detection and quantification of synchrony in real spike trains, as we will see below. Analysis techniques that fall under the general class of ‘jitter methods’ [30] seem to overcome many of the drawbacks of other methods when it comes to detecting synchrony; however, jitter methods have not been used previously to quantify synchrony. Here, I take jitter methods one step further and use them to compute a novel synchrony measure, the Jitter-Based Synchrony Index (JBSI).

In the remainder of this background section, I first show how synchrony can be detected when the two spike trains follow stationary Poisson statistics. I then demonstrate numerically how this detection method fails when firing rates are not stationary and explain how jitter methods overcome this hurdle. Finally, I define and describe two of the synchrony indices commonly employed by experimental neurophysiologists to quantify synchrony. In the Results, I first propose a set of five requirements that an ideal index of synchrony should fulfill, pointing out where existing measures fail to meet these requirements. I then explain how the JBSI is computed and follow with a comparison between the JBSI and previously used indices. Lastly, I demonstrate how the JBSI can be used to estimate the precision of spike timing in the system. Computational details are provided in three appendices.

### Detecting synchrony: the Poisson case

Assume that we have recorded simultaneous spike trains from two neurons. I will refer to the slower-firing neuron as Neuron 1 or the reference neuron, and to the faster-firing neuron as Neuron 2 or the target neuron. Let us denote the number of spikes in the reference and target trains by *n*_*1*_ and *n*_*2*_ and the average firing rates by *r*_*1*_ and *r*_*2*_, respectively (so *n*_*1*_  *n*_*2*_ and *r*_*1*_  *r*_*2*_).

On the face of it, detecting and measuring synchrony seems quite simple. First, decide on a definition of ‘synchrony’: how far apart two spikes can occur and still be considered as synchronous. Throughout this paper, I will use τ_***S***_ to represent this synchrony span. Second, count how many of the spikes fired by the reference neuron occurred within the predefined synchrony span from any spike in the target train. I will denote this observed coincidence count as *N*_*C*_. Third, normalize the coincidence count so it can be used to compare paired spike trains of different lengths. A simple way to do this is to divide it by the total number of reference spikes, transforming the coincidence count to a coincidence rate *R*_*C*_ ≡ *N*_*C*_/*n*_*1*_ (note that rate is used here in the sense of per spike rather than per unit time). The advantage of dividing by *n*_*1*_ rather than, say, *n*_*2*_ or the average of *n*_*1*_ and *n*_*2*_, is that *R*_*C*_ can take values over the full range between 0 (no coincidences) to 1 (all spikes coincident).

On their own, however, *N*_*C*_ and *R*_*C*_ are not very informative, because for any two overlapping series of events there is some non-zero probability that occasionally they will coincide in time, purely by chance. In the case of two spike trains, we want to know whether the two neurons fired independently and the observed coincidences were therefore chance occurrences; this is the null hypothesis. The alternative is that the neurons were coupled, that is, interacted in some way with each other or with a third neuron, causing them to synchronize more (or perhaps less) than expected by chance. Our first task is therefore to determine if the observed coincidence was statistically significant. To do so, we need to know something about the distribution of all chance coincidence counts; if this distribution was known, we could determine the fractional area beyond *N*_*C*_ under the tails of the distribution, in other words, the *p*-value, which would indicate how likely *N*_*C*_ was to belong to this set. At the very least, we would like to know the mean of this distribution - that is, the expected coincidence count **‹***N*_*C*_**› -** and its variance, which would then allow us to calculate a *Z*-score (distance of the observed value from the mean, in units of standard deviation). If the distribution is reasonably close to normal, the *Z*-score can be directly converted to a *p*-value (for example, *p* = 0.05 corresponds to *Z* = ~2 and *p* = 0.001 to *Z* = 3.3).

One way to estimate the distribution of chance coincidences is to assume a specific statistical firing model, and very often the assumption made is that the spike trains are a stationary Poisson process. Under this assumption, the expected number of spikes occurring within an interval ∆*t* is *r·*∆*t*, where *r* is the time-independent (stationary) average firing rate. Going back to our paired spike trains, we observe that, for a sufficiently small synchrony span τ_***S***_, the probability that a reference spike will occur within ***±***τ_***S***_ from any given target spike is equal to the average number of reference spikes in a window of width *2*τ_***S***_, or *2*τ_*S*_*·r*_*1*_. Because the target neuron fired *n*_*2*_ spikes during the recording epoch, we should expect a total of $<N_{C}>=2\tau_{S}\cdot r_{1}\cdot n_{2}$ spike coincidences. For a recording epoch of length *T*, we have *r*_*1*_ *= n*_*1*_*/T* and *r*_*2*_ *= n*_*2*_*/T*, so we can re-write the expected number of coincidences either as:

(1) $$<N_{C}>=2\tau_{S}\cdot r_{1}\cdot r_{2}\cdot T$$

or as:

(2) $$<N_{C}>=2\tau_{S}\cdot n_{1}\cdot n_{2}/T$$

In a Poisson process, the variance of the observed counts is equal to the expected counts: we can therefore test the null hypothesis of independent firing by calculating the likelihood that *N*_*C*_, the observed number of coincidences, belongs to a distribution with a mean and variance of **‹***N*_*C*_**›**.

### Detecting synchrony: when Poisson cannot be assumed

The pitfall in the procedure described above is that, in actuality, we are testing a dual null hypothesis: that (a) the neurons fired independently and that (b) each spike train is a Poisson process with a stationary average firing rate. If (b) happens to be false, we stand the risk of rejecting the null hypothesis, even when (a) is true. We can illustrate this with a simple numerical example.

Assume that both neurons fired 10,000 spikes each during a 250 s recording epoch, so *r*_*1*_ ***=*** *r*_*2*_ = 40 Hz. If τ_*S*_ = 0.5 ms, then the number of coincidences expected by chance is **‹***N*_*C*_**›** = 0.001 × 40 × 40 × 250 = 400, with a standard deviation of 20. Now say that we actually observed 500 coincidences. Because this is five standard deviations above the expected mean, we conclude that this degree of precise synchrony was highly unlikely to be a random deviation (less than one in a million probability) and that the two neurons must have been coupled. Unbeknownst to us, however, both neurons received bursts of inhibitory inputs from a common subset of inhibitory neurons during the recording epoch. These bursts occurred randomly, but were powerful enough to silence both neurons (concurrently) for 20% of the recording epoch. The actual firing epoch (that is, the epoch during which the neurons were ‘allowed’ to fire) was therefore only 200 s, so the firing rate of each neuron was actually 10,000/200 = 50 Hz, and the true expected number of spike coincidences was **‹***N*_*C*_**›** = 0.001 × 50 × 50 × 200 = 500. In other words, the observed synchrony was precisely at chance level. While one could argue that the two neurons were, in a sense, coupled by the common inhibitory input, and thus violated both assumptions (a) and (b), it would clearly be erroneous to conclude that this common inhibitory input caused the neurons to synchronize with a precision of ±0.5 ms.

The example above underscores the fact that it is the ‘local’ firing rate, rather than the global average firing rate, that needs to be taken into account when determining the expected coincidences, since global averaging ignores modulations in firing rates. If these bursts of inhibitory inputs were all identical or nearly so, if they repeated at regular intervals and if we knew their times of occurrence, we could estimate how many coincidences were introduced because of these co-modulations by dividing the full record into segments aligned on the beginning (or end) of each inhibitory burst and exchanging the spike trains of one neuron between segments. The spike coincidences remaining after this procedure could be taken as a good estimate of **‹***N*_*C*_**›** and can then be subtracted from the observed coincidence count *N*_*C*_ to yield an estimate of the ‘excess coincidence’, that is, the coincidence above chance level. This is the same ‘shuffling’ procedure commonly used by electrophysiologists to account for spurious coincidences introduced by a sensory stimulus [31]. However, unlike co-modulations resulting from an experimentally administered stimulus, co-modulations resulting from uncontrolled environmental or systemic influences are rarely reproducible, do not occur at regular intervals and are not locked to the stimulus (if there is a stimulus). Indeed, they may not even be recognized by the experimenter. Therefore, these co-modulations cannot be corrected by shuffling.

Another potential solution is to divide the firing epoch into smaller segments and calculate the local firing rates, and from them the expected coincidences, separately for each segment. This would allow us to correct for any modulations in firing rates that happen on a time scale slower (longer) than the width of each segment. Further scrutiny, however, reveals multiple problems with this approach. First, where should the boundaries between segments be placed? Even a small segment may happen to straddle two epochs with very different firing rates, and may need to be further subdivided. Second, how local is ‘local’ - how small should each segment be? To account for as many co-modulations as possible, some of which may have fast time courses, we would need to make the segments as small as possible; but with very small segments, firing rate estimation would become very imprecise. Ideally, we would like to know the instantaneous firing rate in the immediate vicinity of each spike, but this is impossible to determine from a non-repeating spike train.

The jitter method offers a solution to this dilemma. It allows us to estimate the coincidence count expected from the local firing rate, without explicitly determining the rate itself. To do so, we replace one of the two spike trains with a virtual one (a ‘surrogate’), in which each spike is slightly shifted (or ‘jittered’) from its original time of occurrence by a random amount, within a predetermined ‘jitter window’ of *±*τ_*J*_. This procedure destroys any spike coincidences that may have resulted from interactions on a time scale faster than 2τ_*J*_, but fully preserves local firing rates (and therefore any spike coincidences attributable to co-modulations in these rates) at all slower time scales. Again, we can take the number of jitter-resistant coincidences as an estimator of ‹*N*_*C*_›, the expected number of coincidences, and use it to estimate the excess synchrony. Moreover, if we can determine the distribution of all possible coincidence counts likely to be observed after a jitter, we can determine if the experimentally observed count *N*_*C*_ is an exceptional value (that is, falls in the tails of this distribution) and therefore reflects true, statistically significant synchrony, or if it is likely to be a chance occurrence. Note that the jitter procedure relies on the assumption that co-modulations in firing rates occur on a relatively slow time scale compared with the time scale of the spike coincidences we are interested in. Thus, we need to set τ_*J*_ judiciously: if we set τ_*J*_ too large, we risk destroying some of the chance coincidences caused by co-modulations, and we will then underestimate ‹*N*_*C*_› and overestimate the true degree of synchrony, just as in the numerical example above. Conversely, it would be meaningless to make τ_*J*_ smaller than the synchrony span τ_*S,*_ because such a small jitter would likely preserve all coincidences, both chance and real, and not help us in distinguishing between the two.

The rather intuitive idea of introducing virtual spike jitter was rigorously formulated and explored theoretically and in computer simulations by Geman and colleagues [30,32-34], and applied to experimental data by them [32,35] and others [36-40], in some cases critically [41]. The approach taken here differs in two important ways from these previous implementations. First, it differs in how it determines the distribution of all possible coincidence counts. Previous implementations used the Monte Carlo approach: to generate many (hundreds or thousands of) realizations of jittered spike trains and count the number of spike coincidences for each one, which is computationally intensive. The current approach is based on an analytical computation of the exact probability distribution, thus considerably reducing the computational effort. Even more importantly, previous implementations used the jitter method only to detect synchrony, not to quantify it. Here, I use the jitter method to define a novel synchrony index, the JBSI, which can be used to quantify synchrony and (as we will see) is robust under a wide variety of realistic conditions under which other synchrony indices fail.

### Quantifying synchrony: how to compare different cell pairs

Detecting synchrony is only the first task facing us. Our second task is to quantify the synchrony to allow valid comparisons of synchrony strength between different cell pairs, even when recorded in different experiments or even by different investigators. At first glance, it may seem that the very measures of statistical significance could also be used to quantify the strength of the synchrony. However, measures of statistical significance can be made arbitrarily high (*Z*-scores) or arbitrarily close to zero (*p*-values) by increasing the sample size, that is, by using longer spike trains, even if the rate of synchrony (per spike and per time) remains unchanged. Measures of significance cannot, therefore, be used directly to quantify synchrony - to do so we need a synchrony index.

Many different synchrony indices have been proposed in the literature but relatively few of these have gained popularity with the experimental neurophysiology community, and there seems to be no generally agreed upon ‘best’ index [5,12-14,39]. Neurophysiologists often depict the outcome of paired recordings graphically, by a cross-correlation histogram (cross-correlogram, CCG) [22,31], and most previously employed indices were calculated from the CCG. If the bin width of the raw CCG is chosen to be 2τ_*S*_, then the height of the central bin will be equal to *N*_*C*_. To generate a synchrony index, the coincidence count is first corrected for chance coincidences by subtracting the expected from the observed count; this yields an estimate of excess coincidences. If the spike trains are locked to a repeating stimulus and if repeated spike trains are assumed to be stationary and reproducible, then this correction can be done by subtracting a shuffled cross-correlogram [31]. Otherwise, it is done by subtracting the average number of counts in a region of the CCG equal in width to but away from the central peak. For simplicity, we again assume that the central peak of the CCG is one-bin wide, so that the average count in a far-from-center bin is very close to the overall average count per bin, *b·n*_*1*_*·n*_*2*_*/T*, where *b* is the bin width. With a bin width of 2τ_*S*_ , the average count is therefore equal to our previously defined:

(3) $$<N_{C}>=2\tau_{S}\cdot n_{1}\cdot n_{2}/T$$

Thus, the excess coincidence count is equal to *N*_*C*_***−*‹***N*_*C*_**›**.

Since the excess coincidence count depends on the number of recorded spikes, it is typically normalized in some manner to yield a synchrony index that is not dependent on spike number. For comparison with the JBSI, I selected two of the more commonly used indices in the experimental literature. In the first index, which is usually notated in the literature by E but will be called here Excess Coincidence Index or ECI, the excess coincidence count is simply normalized by the number of spikes in the reference train [42-44]:

(4) $$\text{ECI }\equiv \left(N_{C}-<N_{C}>\right)/n_{1}$$

In the second index, referred to in the literature as the cross-correlation coefficient (CCC) [4,45] or, somewhat loosely, as the correlation coefficient [19,46,47], the excess count is divided by a more complicated expression:

(5) $$CCC\equiv \frac{N_{C}-\langle <,N_{C}^{},>\rangle }{\sqrt{n_{1}\cdot n_{2}\left(1-\frac{n_{1}\cdot b}{T}\right)\left(1-\frac{n_{2}\cdot b}{T}\right)}}$$

This normalization factor is justified mathematically in [48]; for completeness, I derive the CCC from first principles in Appendix C.

Given the availability of these and other synchrony indices in the neurophysiological literature, the reader may wonder: how should an experimenter decide which is the best index to use, and why did the author bother to devise yet one more index? To answer these two questions, I first lay out a set of requirements that, I propose, should be met by an ideal synchrony index. I then show how both the ECI and the CCC fail to meet some of these requirements whereas a novel index, the JBSI, fulfills them all.

## Results and discussion

### What should we expect from an ideal synchrony index?

I propose the following as a minimum set of requirements to be met by an ideal measure of synchrony:

1. The measure should be applicable to both periodic and aperiodic data, and to spontaneous spike trains as well as to stimulated responses. This eliminates from the current discussion a large collection of methods for analyzing periodic signals and measures for quantifying synchrony within the frequency domain (for example, phase coherence) or for quantifying the degree of phase-locking to a precisely repeating stimulus (for example, vector strength) (reviewed in [49-51]).

2. As indicated above, the index should be properly normalized, to allow valid comparison between different experiments. This means that ‘perfect synchrony’ and ‘purely chance synchrony’ should assume the same finite values (say, 1 and 0, respectively), regardless of the experimental details. Several synchrony indices previously used in the literature are un-normalized or improperly so. For example, the synchrony index designated in the literature as k’ [23,52-54] and defined as the ratio *N*_*C*_**/‹***N*_*C*_**›**, is obviously unbounded, because it can assume very large values if **‹***N*_*C*_**›** (the expected coincidence count) is very low.

3. The synchrony measure should reflect the intrinsic strength of the network motif that drives it (common inputs, electrical coupling or mutual inhibition) but should not be sensitive to the mean firing rate, because the latter depends mostly on factors extrinsic to the two neurons such as background synaptic input or experimentally injected current. As previously demonstrated [53,55-57] and as is confirmed in Results, the ECI and several other previously used indices exhibit a strong negative dependence on the firing rate and therefore do not fulfill this requirement.

4. The ideal measure should be maximized whenever spikes fired by Neuron 1 are precisely synchronized with spikes of Neuron 2, even if Neuron 2 fires many more spikes than Neuron 1. As shown in Results and in Appendix C, the CCC is highly sensitive to the firing rate differential between the two trains. Similarly, so-called spike train metrics [58-61] are sensitive to differences between spike times as well as differences between spike numbers and therefore do not meet this requirement.

5. The method should be applicable to any arbitrary pair of concurrent spike trains, without assuming any specific firing statistics or firing rate stationarity. Many published methods for detecting and quantifying synchrony assume, explicitly or implicitly, that (in the case of spontaneous firing) spike trains are stationary Poisson processes (for example, [62]), or, in the case of evoked responses, that responses to repeating stimuli are reproducible (for example, [31,63]). However, real spike trains are, in general, not stationary Poisson processes, as the firing rate is continuously modulated by factors beyond the experimenter’s control or even knowledge. Similarly, repeating stimuli may not generate reproducible responses due to trial-to-trial variation in subject motivation, state of anesthesia, viability of the preparation and so on. These fluctuations will often manifest themselves as modulations in firing rate and/or in latencies, common to both neurons. As previously noted in the literature [64-66] and as illustrated numerically above (see Background), these co-modulations can lead to erroneous detection of correlations or synchrony. I show below that both the ECI and the CCC erroneously indicate synchrony between independently generated spike trains when co-modulations of firing rate are introduced.

### Calculation of the Jitter-Based Synchrony Index

**Figure 1:**
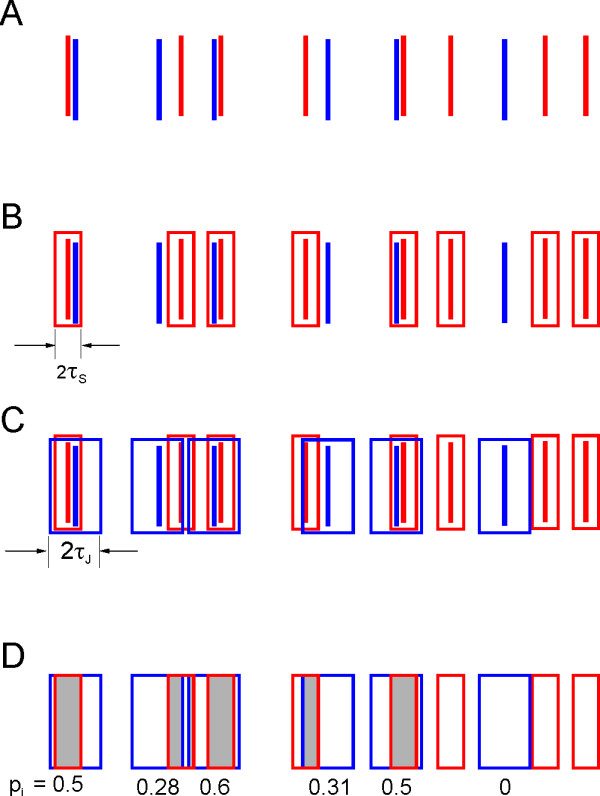
**Using virtual spike jitter to quantify synchrony. (A)** A hypothetical segment from two simultaneously recorded spike trains, with spikes from the reference and target train represented as blue and red bars, respectively. **(B)** By placing a synchrony window (red) of ±τ_S_ centered on each target spike, we see that three of the five reference spikes are synchronous. **(C)** A jitter window (blue) of ±τ_**J**_ is centered on each reference spike. **(D)** Areas of overlap between jitter and synchrony windows are shaded; spikes are omitted for clarity. The probability *p*_*i*_ that any given reference spike will be synchronous after jittering is given by the shaded fraction of its jitter window, and is indicated below the window. The probabilities are then used to determine the expected number of spike coincidences (∑*p*_*i*_), which is then subtracted from the observed number to yield an estimate of excess coincidences. The latter is normalized by the number of spikes in the reference train and multiplied by a scaling factor to yield the JBSI.

Let us assume that we have recorded two simultaneous spike trains. We express each spike train as a vector of spike occurrence times, so we have two vectors: one from the reference neuron, containing *n*_*1*_ spikes designated by their time of occurrence *t*^*1*^_*0*_…*t*^*1*^_***i***_…*t*^*1*^_*n1****-****1*_, and one from the target neuron, containing *n*_*2*_ spikes designated *t*^*2*^_*0*_…*t*^*2*^_***k***_…*t*^*2*^_*n2****-****1*_. We will assume that the time of occurrence of each spike is known to any desirable precision (say 0.1 ms). In Figure 1, reference spikes are depicted in blue and target spikes are depicted in red. The coincidence counting process can be represented graphically by drawing a synchrony window *W*^*S*^_*k*_ of width 2τ_*S*_ centered on each target spike *t*^*2*^_***k***_ (Figure 1B) and counting how many blue spikes fall within a synchrony window. Formally, we assign to each reference spike t_i_^1^ a binary value Syn(i), defined as:

(6) $$Syn\left(i\right)\equiv 1\;if\;t_{i}^{1}\in W^{S}{}_{k}\;\text{for at least one}k;\text{ otherwise }0$$

and then define:

(7) $$N_{C}\equiv \sum_{i}Syn\left(i\right)$$

Obviously, *N*_*C*_ will depend on our choice of the synchrony span τ_*S*_. If τ_*S*_ is increased beyond ½ of the smallest interspike interval in the target train, synchrony windows will begin to overlap, and a given spike in the reference train may be synchronous with more than one spike in the target train. According to (5), it will still only be included once in the coincidence count, thus guaranteeing that *N*_*C*_  *n*_*1*_ or that *R*_*C*_*1*. Also, note that our definition of coincidence does not rely on time binning and thereby avoids the pitfall of two near-coincident spikes falling into two adjacent bins and not being counted as synchronous.

Next, we select a jitter span τ_***J***_, τ_***J***_ > τ_*S*_, and shift (jitter) each reference spike *t*^*1*^_*i*_ by up to ±τ_*J*_. (Note that we jitter only the shorter, reference spike train, thereby conserving computational effort.) As shown below, it is advantageous to choose the ratio τ_*J*_/τ_*S*_ to be 2. The number of coincidences observed after the above jitter procedure, *N*_*C*_^*J*^, is a random variable with some probabilistic distribution; according to our null hypothesis, *N*_*C*_ should be from the same distribution. To test this hypothesis, we need to calculate the probability of observing any given number of total spike coincidences *N*, 0 *≤ N ≤ n*_*1*_, after a jitter. We call this probability *P*^*J*^(*N*):

(8) $$P^{J}\left(N\right)\equiv Pr\left(N_{C}{}^{J}=N\right)$$

To compute *P*^*J*^(*N*), we first calculate the probability *p*_*i*_ that spike *t*^*1*^_*i*_ will be synchronous with spike *t*^*2*^_*k*_, for at least one *k,* after applying the jitter. The process can be represented graphically (Figure 1C) by drawing a jitter window *W*^*J*^_*i*_ of width *2*τ_*J*_, centered on each reference spike *t*^*1*^_*i*_ (blue). It then becomes apparent that *p*_i_ is equal to the fraction of *W*^*J*^_*i*_ that intersects (overlaps) the union set of all synchrony windows (the intersections are shaded in Figure 1D).

Formally:

(9) $$p_{i}\equiv \frac{1}{2\tau_{J}}\cdot \left(W^{J}{}_{i}\cap \underset{k}{U\cup }W^{S}{}_{k}\right)$$

In Appendix A1 we compute the union set of all synchrony windows, and in Appendix A2 we compute *p*_i_. *P*^*J*^(*N*) can then be computed exactly from the vector *p*_i_ using an efficient recursive algorithm, proposed in [67] and provided for the reader’s convenience in Appendix A3. This calculation demonstrates that the distribution *P*^*J*^(*N*) converges rapidly to a normal distribution with the same mean and standard deviation (not shown). We therefore do not need to compute *P*^*J*^(*N*) explicitly to test our null hypothesis - we can use the fact that the fractional area under the tail of a normal distribution, that is, the *p*-value, can be determined directly from the standardized distance of the tail from the mean, the *Z*-score. To calculate the *Z*-score, we only need to know the expected value and the variance of *N*_*C*_^*J*^*,* and these can be derived directly from the *p*_i_’s:

(10) $$\langle N_{C}{}^{J}\rangle =\sum_{i}p_{i}$$

and

(11) $$Var\left(N_{C}{}^{J}\right)=\sum_{i}^{}p_{i}\cdot \left(1-p_{i}\right)$$

(In the case of equal probabilities, *p*_i_ *= p* for all i’s*,* and we get the well-known formulas for the mean and variance of a binomial distribution, *Np* and *Np·(1-p)*, respectively.) The *Z*-score of the experimentally observed synchrony *N*_*C*_ is therefore:

(12) $$Z\equiv \frac{N_{C}-\langle N_{C}{}^{J}\rangle }{\sqrt{Var\left(N_{C}{}^{J}\right)}}=\frac{N_{C}-\sum_{i}p_{i}}{\sqrt{\sum_{i}p_{i}\cdot \left(1-p_{i}\right)}}$$

The *Z*-score tells us if the coincidence count *N*_*C*_ is a statistically significant observation. In other words, it allows us to detect synchrony. However, the *Z*-score cannot be used to quantify synchrony because, like the *p*-value and other measures of statistical significance, it depends on the sample size (the length of the spike train) and therefore is not directly comparable between different experiments. To quantify synchrony in a manner that would allow comparison between experiments, we define a normalized synchrony index - the JBSI:

(13) $$JBSI\equiv \beta \cdot \frac{N_{C}-\langle N_{C}{}^{J}\rangle }{n_{1}}=\beta \cdot \frac{N_{C}-\sum_{i}p_{i}}{n_{1}}$$

where *β* = 2 if τ_*J*_/τ_*S*_ ≤ 2 and *β* = τ_*J*_/(τ_***J***_ − τ_*S*_) if τ_*J*_/τ_*S*_ > 2.

As shown in Appendix B, the scaling factor *β* assures that the JBSI will attain its maximal value of 1 for the case of perfect synchrony. In the case of perfect asynchrony, however, the JBSI will attain its minimal value of –1 only if τ_*J*_/τ_*S*_ ≤ 2 (see Appendix B). In all the simulations below, we select τ_*J*_/τ_*S*_ *=* 2 and therefore *β* = 2.

### Comparison of the Jitter-Based Synchrony Index with cross-correlogram-based synchrony indices

**Figure 2:**
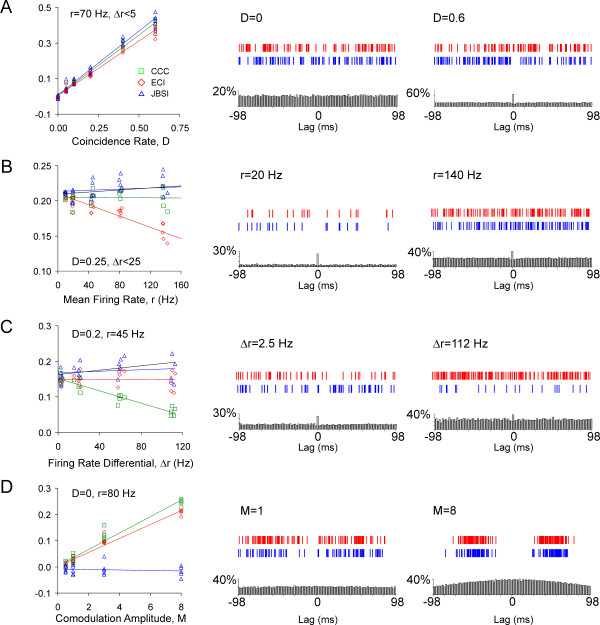
**Comparison of the Jitter-Based Synchrony Index with the Excess Count Index, the cross-correlation coefficient and the corrected Excess Count Index .** In the left column of each panel, the indices and their linear regression lines are plotted for a range of simulations in which one parameter of the simulation was varied. **(A)** The rate of inserted coincidences, *D,* was varied; **(B)** the average firing rate, *r* (defined as the geometrical average of the mean firing rate of the two neurons) was varied; **(C)** the differential in firing rates, *∆r*, was varied; **(D)** the amplitude of firing rate co-modulation, *M*, was varied. In the two rightmost columns, 1 s segments of simulated spike trains, representing two extreme values of the varied parameter, are shown above the cross-correlograms (CCGs) computed from the same trains. CCGs were computed in 2 ms bins and the counts normalized by the number of reference spikes. The black regression lines in B and C represent the corrected Excess Count Index (*ECI*^*cor*^); for clarity, the ECI^cor^ data points are not plotted. In A and D, the ECI^cor^ data points precisely overlapped the CCC. Note that the JBSI was the only index that was robust against variations in all tested parameters.

To test the JBSI and compare it with previously used synchrony indices, I generated simulated paired spike trains that (initially) followed Poisson statistics, by allowing each neuron to fire at random at an average firing rate that could be made time-dependent. Controlled spike coincidences were inserted into each train pair by randomly selecting reference spikes, at a probability *D*, to be shifted to within ±*C* of the next nearest target spike. Thus, the parameter *D* determined the injected coincidence rate (per spike), and the parameter *C* determined the precision of the synchrony (see Methods for details). For the simulations shown in Figure 2, *C* was maintained at 1 ms. The first second of firing from representative simulations is illustrated in the two rightmost columns of Figure 2, with reference and target spikes shown blue and red, respectively. Below each pair of spike trains is the CCG computed from the full train (about 1,000 spikes per neuron) using 2 ms-wide bins. The counts in each bin are normalized by the number of spikes in the reference train, so the height of the central peak is numerically equal to the coincidence rate *R*_*C*_.

I first compared the JBSI with two commonly used synchrony indices, the ECI and the CCC (see Background), by generating simulated spike trains in which the average firing rate in both neurons was maintained constant at about 70 Hz but the rate of injected coincidences *D* was increased parametrically, from 0 to 0.6. For each value of *D*, five runs of the simulation were generated. The three indices and their linear trends are plotted in Figure 2A, left panel. Clearly, all three indices increased more or less linearly with the rate of inserted coincidences, albeit with somewhat different slopes. Thus, under routine conditions, all three indices performed comparably well.

Next, I tested the indices under three challenges. The first challenge was a series of paired spike trains in which *D* was kept constant (at 0.25) while the firing rate in both neurons was parametrically increased from approximately 10 to 140 Hz (Figure 2B). According to requirement three of the set of requirements outlined at the beginning of this section, an ideal synchrony index should be independent of the firing rate. As seen in the left panel of Figure 2B, both the JBSI and the CCC correctly maintained a nearly constant value; in contrast, the ECI trend line dropped steeply with the increased firing rate, incorrectly implying loss of synchrony at the higher firing rates.

Although several previous studies note the negative dependence of the ECI (and other synchrony indices) on the firing rate [53,55-57], they differ in their explanation for this dependency and no remedies are proposed. To see why the ECI is dependent on the firing rate and how this flaw can be corrected, we note that the ECI is meant to estimate the true spike coincidence rate, *R*_*C*_^*true*^, by subtracting the chance spike coincidence rate *R*_*C*_^*chance*^ from the total spike coincidence rate *R*_*C*_. However, the set of chance coincidences and the set of true coincidences are not mutually exclusive sets - their intersection is a set of coincidences that occur at a rate that equals the product of their individual rates, *R*_*C*_^*true*^∙*R*_*C*_^*chance*^. Therefore:

(14) $$R_{C}=R_{C}{}^{\mathrm{true}}+R_{C}{}^{\mathrm{chance}}-R_{C}{}^{\mathrm{true}}\;R_{C}{}^{\mathrm{chance}}$$

This can be solved for *R*_*C*_^*tr****ue***^:

(15) $$R_{C}{}^{\mathrm{true}}=\left(R_{C}-R_{C}{}^{\mathrm{chance}}\right)/\left(1-R_{C}{}^{\mathrm{chance}}\right)$$

We should therefore correct the definition of the ECI by dividing it by (1- *R*_*C*_^*chanc****e***^). Since *R*_*C*_^*chanc****e***^ = ***‹****N*_*C*_***›/****n*_*1*_, the corrected ECI, or ECI^cor^, is:

(16) $${\text{ECI}}^{\mathrm{cor}}\equiv \left(N_{C}-<N_{C}>\right)/\left(n_{1}-<N_{C}>\right)$$

Note that in the case of the JBSI such a correction is not needed, because chance spike coincidences are defined as spike coincidences that survive the jitter process, while true spike coincidences are those that are destroyed by the jitter process, and these two sets are mutually exclusive. As shown in Figure 2B (black trend line), the ECI^cor^ was numerically very close to the JBSI and was not dependent on the firing rate.

The second challenge tested the fourth of the requirements outlined at the beginning of this section, that the index be independent of any firing rate differential between the two neurons. This was tested by simulated spike trains in which the injected coincidence rate *D* was maintained at 0.2, the geometric mean of the two average firing rates (*r*) was kept at approximately 45 Hz, but the difference between the two firing rates (∆*r*) was parametrically increased from 2.5 to 110 Hz (Figure 2C). Both the JBSI and the ECI correctly maintained a nearly constant value, and so did the ECI^cor^ (black trend line), but the CCC decreased steeply with ∆*r*.

The dependence of the CCC on the firing-rate differential is a direct result of its definition. As shown in Appendix C, for a fixed number of spikes per train, the value of the CCC for the case of perfect synchrony, CCC_max_, will not attain 1 unless the number of spikes in the two trains is equal; otherwise, CCC_max_ will be <1 and will decrease with an increasing firing rate differential. This suggests a simple remedy - divide the CCC by CCC_max_ to yield a corrected CCC index, the CCC^cor^. Interestingly, as shown in Appendix C, the CCC^cor^ turns out to be identical to the ECI^cor^ above. Thus, the ECI^cor^ (or CCC^cor^) satisfies both requirements three and four, and is therefore preferable to either the ECI or the CCC. Indeed, when firing rates are stationary, the ECI^cor^ may be the index of choice because it has all the advantages of the JBSI but is much easier to compute.

Unfortunately, the ECI^cor^ fails, as do the ECI and the CCC, in regards to requirement five, in that these indices are sensitive to co-modulations in the firing rates. This is illustrated in Figure 2D, which represents simulations with independent spike trains (that is, *D* = 0) in which the total number of spikes was maintained nearly unchanged at about 1,000 per train but the mean firing rate of both neurons was co-modulated in time with a period of 0.5 s, with the amplitude of the modulating waveform varied parametrically. Even though no coincidences were inserted into these trains, the values of the ECI and the CCC were > 0 and increased with the modulation amplitude (note that the ECI^cor^ is not plotted in Figure 2D, because in these simulations the firing rate differential was very low, so the ECI^cor^ was virtually identical to the CCC). This is a fatal, non-remediable flaw in these commonly used synchrony indices, originating in the basic assumption that the two spike trains are a Poisson process, an assumption that is violated (as we saw in Background) when co-modulations are present. In contrast, the JBSI correctly reported no synchrony for these independent spike trains, even when co-modulations were present.

### Comparison of the Jitter-Based Synchrony Index with the Jitter-Sensitive Synchrony Index

In a previous experimental publication [28] we introduced the Jitter-Sensitive Synchrony Index (JSSI) and used it to quantify sub-millisecond firing synchrony between inhibitory cortical interneurons. The JSSI was defined as the *Z*-score of the observed coincidence count (Equation 11) normalized by *((*τ_*J*_/τ_*S*_*-1)·n*_*1*_*)*^*1/2*^. Like the JBSI, the JSSI is robust in regards to co-modulations in firing rates; however, the JSSI exhibits a negative dependency on the overall firing rate and therefore the JBSI is preferable to the JSSI.

### Estimating the temporal precision of neuronal firing

**Figure 3:**
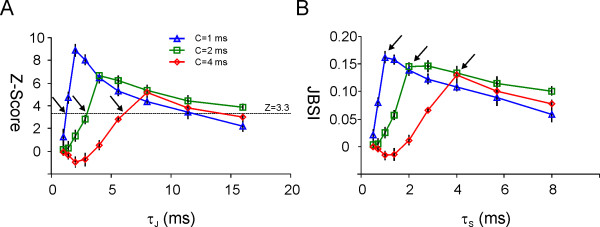
**Estimating temporal precision of firing using the Jitter-Based Synchrony Index.** The three superimposed plots represent different simulated paired spike trains; each plot is an average of five runs of the simulation (error bars indicate standard error of the mean). The average firing rate *r* (approximately 45 Hz) and the rate of inserted coincidences D (0.2) were identical for all pairs, but the precision of synchrony C was varied from 1 to 4 ms. **(A)** The *Z*-score is plotted against the jitter span τ_*J*_, which was varied from 1 to 16 in √2-fold increments while τ_*S*_ was maintained at τ_*J*_/2. The intersections of the three plots with the line *Z* = 3.3, representing a significance threshold of *p* = 0.001, correspond to τ_*J*_ = 1.5, 3 and 6 ms (black arrows). **(B)** For the same simulations as in (A), the JBSI is plotted for increasing values of the synchrony span τ_*S*_. Cutoff points, from which the JBSI fell steeply to the left, correspond to τ_*S*_ = 1, 2 and 4 ms (black arrows).

How precise is neuronal firing in the brain, and how does one measure this precision? These questions have long occupied both experimental and theoretical neuroscientists, and do not yet have a satisfactory answer [7,68-71]. Jitter methods are particularly amenable for testing hypotheses regarding temporal precision. Jitter methods test a specific null hypothesis: that the temporal precision of firing is no better than *±*τ_*J*_, for any desired τ_*J*_, and therefore a jitter of up to *±*τ_*J*_ should not reduce the observed coincidence count [30,32-34]. If the probability that the null hypothesis is true falls below any predetermined threshold, one can conclude that firing precision was better than *±*τ_*J*_. One can then proceed to test increasingly smaller values of τ_*J*_ until the null hypothesis can no longer be rejected; the smallest value of τ_*J*_ that allows rejection of the null hypothesis can be considered an estimate of the firing precision in the system. This is illustrated in Figure 3A, in which three simulated paired spike trains were used to calculate *Z*-scores and JBSI for τ_*J*_ values increasing from 1 to 16 ms in multiples of √2, while maintaining τ_*J*_/τ_*S*_ = 2. The three superimposed plots correspond to simulations with the same rate of inserted coincidences (determined by the parameter *D,* which for these simulations was fixed at 0.2) but with different degrees of precision of synchrony (determined by the parameter *C*, which was varied between 1, 2 and 4 ms, as indicated in the figure legend). Each plot is an average of five different runs of the simulation. The black arrows in Figure 3A point to the intersection of the graphs with the line Z = 3.3, corresponding to *p* = 0.001. With this stringent threshold for significance, firing precision was better than about 1.5, 3 and 6 ms, respectively, for the three simulations, consistent with the corresponding precision parameter *C* used to generate each simulation.

The problem with this approach is the arbitrariness of any selected significance threshold and the fact (already alluded to in this article) that one can increase statistical significance at will, simply by recording longer spike trains. To remove the dependence on the length of the train, one can use the JBSI instead of the *Z*-score, as is done in Figure 3B; however, the question then becomes what JBSI value one should use as a threshold. The solution is to determine firing precision based on the shape of the JBSI versus τ_*S*_ curve (Figure 3B) rather than on any particular threshold. As illustrated in Figure 3B, this curve is strongly asymmetric, sloping shallowly from its peak value rightwards, but sloping steeply to the left. It is instructive to examine the reason for these two different slopes. The shallow rightward decrease in JBSI values reflects the gradual increase in the widths of the synchrony windows *W*^*S*^: with the union of all synchrony windows occupying a larger fraction of the spike record, a jittered spike is more likely to fall into a synchrony window and thereby preserve or increase the expected coincidence count ‹*N*_*C*_^*J*^›. The steep decline to the left results from a very different reason - it reflects the jitter parameter τ_*J*_ falling to values that are too small to make any difference in the temporal structure of the spike train; in other words, τ_*J*_ falling below the temporal precision of the system. This suggests that one should use the points at which the curves drop off steeply to the left as cutoff points for determining firing precision. The cutoff points indicated by arrows in Figure 3B correspond to τ_*S*_ values of 1, 2 and 4 ms, exactly the values of the corresponding precision parameter *C*.

### Other applications of the Jitter-Based Synchrony Index method

In this manuscript I demonstrate the utility of the JBSI method for quantifying precise firing synchrony between two neurons; in principle, however, this method can be extended to other temporal relationships between spike trains. For example, instead of testing for synchrony (zero lag between the two spikes), one can test for a recurring lag *LL* ≠ 0, between the two spike trains, such as postulated by the ‘synfire chain’ hypothesis [46]. This is equivalent to shifting the red synchrony windows in Figure 1B by *L.* One can also use the JBSI to measure precision of firing between recurring trials recorded from a single neuron in response to a repetitive stimulus, by regarding the stimulus as the reference train. To generate a time-resolved index of synchrony, one can calculate the JBSI over a sliding window of any chosen width, provided that the firing rate is high enough to generate sufficient spikes within each window for a reliable computation. Finally, although a bivariate measure in its present form, the JBSI can be extended to synchrony between multiple simultaneously recorded spike trains, for example by averaging over all possible pairs. Future work may generalize the JBSI to a truly multivariate measure of synchrony.

## Conclusions

I describe here a conceptually simple and computationally efficient method for determining the statistical significance of firing synchrony between two neurons and for quantifying synchrony as a normalized index, the JBSI. The method is based on the introduction of virtual spike jitter, but unlike previous implementations of this idea, it does not rely on computationally intensive generation of surrogate spike trains, and it uses jitter not only to test the statistical significance of spike coincidences but also to quantify synchrony. To evaluate the JBSI in comparison with previously used synchrony measures, I propose a set of five requirements from an ideal measure of synchrony. I show that the JBSI meets them all, unlike some commonly used synchrony indices such as the ECI and the CCC. First, the JBSI can be computed for any pair of spike trains, whether spontaneous or locked to a repeating stimulus and whether periodic or not. Second, the JBSI is well-normalized, in that it assumes values between 1 (highest possible synchrony for a given number of spikes in each train) and −1 (lowest possible synchrony), with 0 indicating chance-level synchrony. Third, the JBSI is independent of the firing rate, whereas the ECI is not. Fourth, the JBSI is independent of the firing rate differential between the two neurons, whereas the CCC is not. Finally, the JBSI is robust against co-modulations in firing rate, while both the ECI and the CCC show spurious synchrony when such co-modulations are present. I also show that a minor modification in the definitions of the ECI and the CCC results in an improved index, the ECI^cor^, which is robust under the third and fourth requirements and is therefore superior to both the ECI and the CCC. The ECI^cor^ may indeed be the index of choice due to its computational simplicity, if firing rates are known to be stationary. However, the JBSI is the only index that meets all five requirements. By virtue of its robustness, the JBSI can be used to compare firing synchrony between experiments conducted under widely different experimental conditions and, as I demonstrate, it can also be used to estimate the temporal precision of firing in the system.

## Methods

All computations and simulations were implemented in MathCad (PTC); the MathCad code is available from the author upon request. A MatLab routine for calculating the JBSI will be made available on the journal website.

### Spike train simulations

For most simulations, the epoch length and/or the firing rate were adjusted so the generated trains consisted of about 1,000 spikes each. Each simulation started by generating two independent Poisson spike trains, as follows: for each 1 ms bin of the time epoch, a spike was considered fired if a randomly generated number between 0 and 1 was smaller than the predetermined firing rate (in spikes/ms); the precise time of the spike within the 1 ms bin was then randomly determined. To enforce a refractory period of 2 ms, if a spike was fired then no spikes were allowed in the next two bins. Next, firing coincidences, at a rate *D* and a precision *C*, were inserted as follows: for each spike of the reference train, if a randomly generated number was smaller than *D*, the spike was shifted forward to within ±*C* of the next spike of the target train. Finally, any spike in the shifted reference train that violated the refractory period was removed; typically, this resulted in the final spike count of the two spike trains differing by about 5% to 10% (if the initial, predetermined firing rates were equal). To generate co-modulations in the firing rate (Figure 2D), the predetermined firing rate was multiplied by a rectified sinusoidal function with a period of 1 s, raised to the power *M*: *M* was parametrically increased to vary the depth of the modulation.

### Appendix A

#### Algorithm for calculating the union set of all synchrony windows

Given the vector *t*^*2*^ representing the target spike train and the selected synchrony span τ_*S*_, this algorithm returns a *d* by *2* matrix *U*, with each of the *d* rows representing a contiguous time segment [U_m,0_, U_m,1_], *0* ≤ *m* ≤ *(d-1)*.

(17) $$\begin{array}{l} U_{0,0}=t^{2}{}_{0}-\tau_{S}\\ \begin{array}{cc} U_{0,1}=t^{2}{}_{0}+\tau_{S} & if\;t^{2}{}_{0}+\tau_{S}<t^{2}{}_{1}-\tau_{S}\\ otherwise\;t^{2}{}_{1}+\tau_{S} & if\;t^{2}{}_{1}+\tau_{S}<t^{2}{}_{2}-\tau_{S}\\ otherwise\;t^{2}{}_{2}+\tau_{S} & etc\text{.} \end{array} \end{array}$$

#### Algorithm for computing the probability vector p_i_

(18) $$p_{i}\equiv \frac{1}{2\tau_{J}}\cdot \left(W^{J}{}_{i}\cap \underset{k}{\cup }W^{S}{}_{k}\right)$$

The union of all synchrony windows, $\underset{k}{\cup }W^{S}{}_{k}$ , is first expressed as the *d by 2* matrix *U* (Appendix A1). Next we calculate *I*_*i,m*_, the intersection of the jitter window *W*^*J*^_*i*_ with the segment [*U*_*m,0*_, *U*_*m,1*_], as follows (*t*^*1*^ is the vector representing the reference spike train):

(19) $$\begin{array}{cc} I_{i,m}=0 & if(U_{m,0}-\tau_{J})\geq t^{1}{}_{i}\;or(U_{m,1}+\tau_{J})\leq t^{1}{}_{i}\\ \tau_{J}-\left(U_{m,0}-t^{1}{}_{i}\right) & if\left(U_{m,0}-\tau_{J}\right)\leq t^{1}{}_{i}\leq min\left(U_{m,0}+\tau_{J}\text{,}\;U_{m,1}-\tau_{J}\right)\\ \tau_{J}+\left(U_{m,1}-t^{1}{}_{i}\right) & if\left(U_{m,1}+\tau_{J}\right)\geq t^{1}{}_{i\;}\geq max\left(U_{m,0}+\tau_{J}\text{,}\;U_{m,1}-\tau_{J}\right)\\ min\left(U_{m,1}-U_{m,0}\text{,}\;2\tau_{J}\right) & otherwise \end{array}$$

Note that if τ_*S*_ is smaller than ½ the smallest target interspike interval and therefore synchrony windows do not overlap, we have *U*_*k,0*_ *= t*^*2*^_*k*_*-* τ_*S*_ and *U*_*k,1*_ *= t*^*2*^_*k*_ *+* τ_*S*_, and (A2.1) simplifies to

(20) $$\begin{array}{l} \begin{array}{cc} I_{i,k}=0 & \;if\;{|t}^{1}{}_{i}–t^{2}{}_{k}|\geq \tau_{J}+\tau_{S} \end{array}\\ \begin{array}{cc} 2\tau_{S} & \;if|t^{1}{}_{i}-t^{2}{}_{k}|\leq \tau_{J}-\tau_{S} \end{array}\\ \begin{array}{cc} \left(\tau_{J}+\tau_{S}-|t^{1}{}_{i}-t^{2}{}_{k}|\right) & otherwise \end{array} \end{array}$$

Finally, we sum over all *m* segments and divide by the width of the jitter window to yield *p*_*i*_:

(21) $$p_{i}=\frac{1}{2\tau_{J}}\cdot \sum_{m}I_{i,m}$$

#### Algorithm for calculating the probability of *N* successes in *n*_*1*_ trials with non-homogeneous success probabilities (adapted from [67])

We regard the reference spike train after a jitter as a Bernoulli series of *n*_*1*_ trials indexed on *i*, each with its own success (that is, synchrony) probability *p*_*i*_ (calculated in Appendix A2) and failure probability *q*_*i*_ = *1− p*_i_. We calculate the probability *P*^*J*^*(N)*, 0 *≤ N ≤ n*_*1*_, that exactly *N* of the spikes will be synchronous, by constructing a triangular *n*_*1*_ by (*n*_*1*_ *+ 1)* matrix *P* recursively. The first three rows are:

(22) $$\begin{array}{cccc} P_{1,0}=q_{0} & P_{1,1}=p_{0} & & \\ P_{2,0}=q_{0}q_{1} & P_{2,1}=q_{0}p_{1}+p_{0}q_{1} & P_{2,2}=p_{0}p_{1} & \\ P_{3,0}=q_{0}q_{1}q_{2} & P_{3,1}=q_{0}q_{1}p_{2}+q_{0}p_{1}q_{2}+p_{0}q_{1}q_{2} & P_{3,2}=q_{0}p_{1}p_{2}+p_{0}q_{1}p_{2}+p_{0}p_{1}q_{2} & P_{3,3}=p_{0}p_{1}p_{2} \end{array}$$

We define *P*_*k,j*_ = 0 for *j > k*.

Inspection shows that each row can be expressed in terms of the previous row:

(23) $$P_{m,M}=p_{m-1}\cdot P_{m-1,M-1}+q_{m-1}\cdot P_{m-1,M}$$

The final row gives us the desired probability, *P*^*J*^*(N) = P*_*n1,N*_*.*

### Appendix B

#### A proof that the Jitter-Based Synchrony Index is bounded between −1 and 1

In the discussion below, the ratio of the jitter to synchrony windows will be indicated by α, that is, α = τ_***J***_**/**τ_*S*_. The JBSI will be maximized when ‹*N*_*C*_^***J***^›, that is, the expected coincidence count following a jitter, is minimized. This will happen when each of the spikes of Neuron 1 is at least τ_***J***_ ***+*** τ_*S*_ away from any spike of Neuron 2 other than spikes it is synchronized with, and is infinitesimally less than τ_*S*_ away from the spike it is synchronized with. Note that if one constructs a cross-correlogram of the two spike trains with a bin width of 2τ_*S*_ and the central bin symmetric about 0, the first condition is equivalent to saying that the two off-center bins should have 0 counts. Under these optimal conditions, the probability that a synchronous spike will remain synchronous after a random jitter is ½ for α 2 or 1/α for α ≥ 2, and *P*^*J*^*(N)* becomes a binomial distribution with the binomial parameters *p* = *1/*α and *N = N*_*C*_*.* A binomial distribution has an expected value of *N·p*, so for α ≥ 2 the JBSI becomes:

(24) $$JBSI=\frac{\alpha }{\alpha -1}\cdot \frac{N_{C}-N_{C}\cdot \frac{1}{\alpha }}{n_{1}}=\frac{\alpha }{\alpha -1}\cdot \frac{N_{C}\left(1-\frac{1}{\alpha }\right)}{n_{1}}=\frac{N_{C}}{n_{1}}=R_{C}$$

It is easily verified that the same holds for α ≤ 2.

In other words, in this optimal scenario, JBSI is equal to the observed coincidence rate *R*_*C*_. Since by definition *R*_*C*_1, we have JBSI 1.

The JBSI can also attain negative values, indicating less-than-expected synchrony (not to be confused with ’anti-synchrony’, which is usually meant to indicate precise out-of-phase relationships). For example, if none of the spikes are synchronous but some of the reference spikes are within τ_***J***_ ***+*** τ_*S*_ of target spikes (that is, the center bin in the CCG is 0, but the off-center bins are not), there is a non-zero probability that some spikes will become synchronous after a jitter. This will render both the *Z*-score and the JBSI negative. To calculate the lowest possible values of the JBSI, we look at the extreme case in which each reference spike is infinitesimally more than τ_*S*_ away from some target spike, and therefore has a probability of *1/*α (1/2 for α2264 2) of becoming synchronous after a jitter. Again we have a binomial distribution, yielding JBSI = −1 for α ≤ 2, or JBSI = −1*/(*α*-1)* for α ≥ 2. It is therefore both numerically convenient and advantageous to select α = 2, as this ratio will allow the maximal dynamic range for the JBSI.

### Appendix C

#### Derivation of the Cross-Correlation Coefficient

Assume that we have recorded two simultaneous spike trains, during a recording epoch of duration *T*, with *n*_*1*_ and *n*_*2*_ spikes each, respectively. Assume *n*_*1*_ ≤ *n*_*2*_. We would like to calculate the probability *P(N)* of observing exactly *N* coincidences, 0 ≤ *N* ≤ *n*_*1*_. The CCC is based on the assumption that the two spike trains are independent and that the spikes occur randomly in time. We bin the epoch *T* into *K* bins, with bin width small enough so no more than one spike of each neuron can occur per bin. We can recreate the two spike trains in the following manner. First, we generate train #2, by distributing *n*_*2*_ spikes at random into *n*_*2*_ bins. We then generate train #1 by distributing the *n*_*1*_ spikes into *n*_*1*_ bins in the same random manner. What is the probability that *N* of these *n*_*1*_ bins already contain a spike from train #2? This can be solved by basic combinatorics, as follows. There are $\left(\begin{array}{c} n_{2}\\ N \end{array}\right)$ distinct configurations of *N* objects (spikes, in our case) in *n*_*2*_ bins, where $\left(\begin{array}{c} Y\\ X \end{array}\right)\equiv \frac{Y!}{X!\left(Y-X\right)!}$ is the binomial coefficient. For each of these configurations, there are $\left(\begin{array}{c} K-n_{2}\\ n_{1}-N \end{array}\right)$ ways to place the remaining (*n*_*1*_*-N*) objects in the remaining (*K-n*_*2*_) bins, so in total there are $\left(\begin{array}{c} n_{2}\\ N \end{array}\right)\cdot \left(\begin{array}{c} K-n_{2}\\ n_{1}-N \end{array}\right)$ distinct configurations with exactly N spikes from train #1 falling into bins that already contain a spike from train #2. To convert this number into probability, we need to divide by the total number of possible configurations of *n*_*1*_ objects in *K* bins, which is $\left(\begin{array}{c} K\\ n_{1} \end{array}\right)$ . The requested probability is therefore:

(25) $$P\left(N\right)=\frac{\left(\begin{array}{c} n_{2}\\ N \end{array}\right)\cdot \left(\begin{array}{c} K-n_{2}\\ n_{1}-N \end{array}\right)}{\left(\begin{array}{c} K\\ n_{1} \end{array}\right)}$$

This is the well-known hypergeometric distribution, that has a mean (expected coincidences count) and a variance given, respectively, by:

(26) $$E_{\mathrm{HG}}=\frac{n_{1}n_{2}}{K}$$

and

(27) $$VAR_{\mathrm{HG}}=\frac{n_{1}n_{2}}{K-1}\left(1-\frac{n_{1}}{K}\right)\left(1-\frac{n_{2}}{K}\right)$$

The CCC is defined as the *Z*-score of the observed coincidence count under the assumption of a hypergeometric distribution, normalized by √(*K*-1):

(28) $$CCC\equiv \frac{N_{C}-E_{\mathrm{HG}}}{\sqrt{\left(K-1\right)VAR_{\mathrm{HG}}}}=\frac{N_{C}-\frac{n_{1}\cdot n_{2}}{K}}{\sqrt{n_{1}\cdot n_{2}\left(1-\frac{n_{1}}{K}\right)\left(1-\frac{n_{2}}{K}\right)}}$$

To verify that this expression is indeed normalized, we substitute for *N*_*C*_ the highest possible coincidence count, which is the number of spikes in the shorter train, *n*_*1*_:

(29) $$\begin{array}{l} CCC_{\max}=\frac{n_{1}\cdot \left(1-\frac{n_{2}}{K}\right)}{\sqrt{n_{1}\cdot n_{2}\left(1-\frac{n_{1}}{K}\right)\left(1-\frac{n_{2}}{K}\right)}}\\ =\sqrt{\frac{n_{1}\cdot (1-\frac{n_{2}}{K})}{n_{2}\left(1-\frac{n_{1}}{K}\right)}}=\sqrt{\frac{\frac{K}{n_{2}}-1}{\frac{K}{n_{1}}-1}} \end{array}$$

Clearly, for all *n*_*1*_  *n*_*2*_, we will have CCC_max_1, so the CCC is normalized. However, CCC as defined will only reach 1 if *n*_*1*_ = *n*_*2*_, that is, if the two firing rates are equal. This is a disadvantage compared to the JBSI, which will be 1 for perfectly synchronized trains, even if the two spike trains have very different rates. This suggests a simple way to correct the CCC -divide it by the CCC_max_:

(30) $$\begin{array}{l} \;CCC^{\mathrm{cor}}=\frac{N_{C}-E_{\mathrm{HG}}}{\sqrt{n_{1}\cdot n_{2}\left(1-\frac{n_{1}}{K}\right)\left(1-\frac{n_{2}}{K}\right)}}\cdot \sqrt{\frac{n_{2}\cdot \left(1-\frac{n_{1}}{K}\right)}{n_{1}\left(1-\frac{n_{2}}{K}\right)}}\\ =\frac{N_{C}-\frac{n_{1}\cdot n_{2}}{K}}{n_{1}\cdot \left(1-\frac{n_{2}}{K}\right)}\;\\ =\frac{N_{C}-\frac{n_{1}\cdot n_{2}}{K}}{n_{1}-\frac{n_{1}\cdot n_{2}}{K}} \end{array}$$

If we choose the bin width to be *2*τ_*S*_, then *K* = *T*/*2*τ_*S*_, and therefore (using equation 2) *n*_*1*_*·n*_*2*_*/K* = *‹N*_*C*_*›*

So, using equation 14:

(31) $${\text{CCC}}^{\mathrm{cor}}=\left(N_{C}-<N_{C}>\right)/\left(n_{1}-<N_{C}>\right)={\text{ECI}}^{\mathrm{cor}}$$

## Abbreviations

CCC, cross-correlation coefficient; CCCcor, corrected CCC; CCG, cross-correlogram; ECIcor, corrected Excess Count Index; ECI, Excess Count Index; JBSI, Jitter-Based Synchrony Index; JSSI, Jitter-Sensitive Synchrony Index.

## Competing interests

The author declares no competing financial interests.
